# A Distributed Learning Method for $\ell_{1}$-Regularized Kernel Machine over Wireless Sensor Networks

**DOI:** 10.3390/s16071021

**Published:** 2016-07-01

**Authors:** Xinrong Ji, Cuiqin Hou, Yibin Hou, Fang Gao, Shulong Wang

**Affiliations:** 1 Beijing Engineering Research Center for IOT Software and Systems, Beijing 100124, China; jixinrong@emails.bjut.edu.cn (X.J.); houcuiqin@bjut.edu.cn (C.H.); gaofang@emails.bjut.edu.cn (F.G.); wangshulong@emails.bjut.edu.cn (S.W.); 2 School of Information & Electrical Engineering, Hebei University of Engineering, Handan 056038, China

**Keywords:** kernel machines, distributed learning, *ℓ*_1_ norm regularization (*ℓ*_1_-regularized), kernel minimum mean squared error (KMSE), wireless sensor network (WSN)

## Abstract

In wireless sensor networks, centralized learning methods have very high communication costs and energy consumption. These are caused by the need to transmit scattered training examples from various sensor nodes to the central fusion center where a classifier or a regression machine is trained. To reduce the communication cost, a distributed learning method for a kernel machine that incorporates $\ell_{1}$ norm regularization ($\ell_{1}$-regularized) is investigated, and a novel distributed learning algorithm for the $\ell_{1}$-regularized kernel minimum mean squared error (KMSE) machine is proposed. The proposed algorithm relies on in-network processing and a collaboration that transmits the sparse model only between single-hop neighboring nodes. This paper evaluates the proposed algorithm with respect to the prediction accuracy, the sparse rate of model, the communication cost and the number of iterations on synthetic and real datasets. The simulation results show that the proposed algorithm can obtain approximately the same prediction accuracy as that obtained by the batch learning method. Moreover, it is significantly superior in terms of the sparse rate of model and communication cost, and it can converge with fewer iterations. Finally, an experiment conducted on a wireless sensor network (WSN) test platform further shows the advantages of the proposed algorithm with respect to communication cost.

## 1. Introduction

A wireless sensor network (WSN) consists of a large number of small battery-powered devices that can sense, process and communicate data. WSNs are used on a wide scale to monitor the environment, track objects, and so on [1,2]. Classification and regression of monitoring information are the most fundamental and important tasks in a variety of WSN applications such as vehicle classification, fault detection and intrusion detection [3,4,5]. Therefore, many machine learning methods developed for classification or regression problems are increasingly widely used in WSNs [6,7]. However, WSNs are frequently characterized as networks with a central node that runs main operations such as network synchronization, data processing and storage, while the remaining nodes only obtain and transmit information to the central node. For machine learning problems in WSNs, the scattered training examples must be transmitted from different sensor nodes to the central fusion center by multi-hop routing. Then, all the training examples are used at the central fusion center to train a classifier or a regression machine using the batch learning method. In this paper, this learning method is referred to as the “centralized learning method”. Therefore, centralized learning methods have very high communication costs and energy consumption and are liable to cause congestion and failure on nodes near the central fusion center. This will lead to an energy imbalance among the nodes and greatly reduce the lifetime of the WSN [8]. To avoid and solve these problems, distributed learning methods for classifiers or regression machines, which depend on in-network processing through collaboration between single-hop neighboring nodes, have attracted more and more interest from researchers [9,10,11,12,13,14,15,16,17].

The kernel method or kernel machine, which is shorthand for the machine learning method based on the kernel function, has attracted broad attention because of the successful application of support vector machines (SVMs) and statistical learning theory. Because of the incomparable advantages in solving nonlinear problems, the kernel method has been successfully applied to many fields and has become a mainstream method of machine learning [18,19,20,21,22,23]. As WSN applications become more widespread, research on distributed learning methods for the kernel machine have attracted increasing attention in recent years [24,25,26,27]. Guestrin et al., in [10], presented a general framework for a distributed linear regression and proposed a distributed learning method that depends on the fixed network structure. Unfortunately, this method requires a very high computational overhead to maintain the network structure, and it does not apply to nonlinear kernel machines. Predd et al., in [11,12], provided a distributed collaborative learning algorithm for a linear kernel regression machine that depends on a collaboration between single-hop neighboring nodes and a consistency constraint on the prediction value of each shared training example. Because of the dependence on shared training examples, the convergence, convergence rate and communication cost of this algorithm are greatly affected by the number and distribution of shared training examples. Forero et al., in [13], proposed a distributed learning method for linear SVMs based on a consensus of weights and biases between single-hop neighboring nodes. Then, Forero et al., in [14], presented a distributed learning method for nonlinear SVMs that depends on sharing the training examples among all nodes and constrains the consensus on the prediction of shared training examples on all nodes. However, the construct of shared training examples is very cumbersome in this method. Flouri et al., in [15,16], and Yumao et al., in [17], proposed distributed learning methods for SVM based on the sparse characteristic of SVM. Because the sparse characteristic of SVM is determined by the hinge loss function, the available distributed learning algorithms for SVMs in WSNs still have a very high communication cost.

As an extension to the minimum mean squared error, the kernel minimum mean squared error (KMSE) was developed for solving nonlinear classification or regression problems. Its excellent performance and general applicability are well known and have been demonstrated [28]. Moreover, $\ell_{1}$ norm regularization ($\ell_{1}$-regularized) is widely used to solve optimization problems by incorporating an $\ell_{1}$ norm penalty, and it can identify parsimonious models for training examples such as Lasso and Compressive Sensing [29]. To solve the problems of dependence on a fixed network structure and shared training examples and the high communication cost in existing distributed learning methods for kernel machines, this paper introduces the $\ell_{1}$-regularized term instead of the $\ell_{2}$-regularized term to construct the optimization problem of KMSE and obtain a sparse model that can reduce the amount of data transmitted between neighboring nodes. Therefore, this paper proposes a distributed learning algorithm for the $\ell_{1}$-regularized KMSE estimation problem that depends on in-network processing and collaboration by transmitting the sparse model only between single-hop neighboring nodes and is independent of the shared training examples and the fixed network structure. Simulation results demonstrate that the proposed algorithm can obtain an almost identical prediction accuracy as that obtained by the batch learning method, has significant advantages in terms of the sparse rate of model and communication cost compared with the existing algorithms and can converge with relatively few iterations.

The remainder of this paper is organized as follows. In Section 2, we briefly review the supervised learning model of the KMSE estimation problem, discuss the alternating direction method of multipliers, and describe the problem to be solved in this paper. In Section 3, we present a detailed derivation and solution for the $\ell_{1}$-regularized KMSE estimation problem and the collaborative approach between neighboring nodes. Then, we describe the proposed distributed learning algorithm for the $\ell_{1}$-regularized KMSE estimation problem. In Section 4, we evaluate the performance of the proposed algorithm by extensive simulation experiments with both synthetic dataset and datasets from UC Irvine Machine Learning Repository (UCI). In Section 5, we conduct an experiment on a WSN test platform to further validate the performance of the proposed algorithm with regard to communication cost. Finally, we conclude the paper in Section 6.

## 2. Preliminaries and Problem Statement

In this section, we briefly review the supervised learning model of KMSE estimation problems, discuss the alternating direction method of multipliers, and then describe the problem to be addressed in this paper.

### 2.1. Kernel Minimum Mean Squared Error Estimation

Given a training example set $S=\{(x_{i},y_{i})\},\;x_{i}\in {ℜ}^{d},\;i=1,...,m$, where $y_{i}\in \{1,-1\}$ or $y_{i}\in ℜ$ are drawn independently and identically distributed from an unknown distribution, a functional relationship between the inputs x and the outputs y can be inferred that minimizes the mean squared error between the outputs y and the predictions f(x). According to the empirical risk minimization principle that can directly estimate the function f(⋅) from the training examples, a function f(⋅) is selected from a class of functions that minimizes the empirical risk as shown in Equation (1):

(1) $$R_{emp}(f)=\frac{1}{m}\sum_{i=1}^{m}{\left(y_{i}-f(x_{i})\right)}^{2}$$

Kernel methods are used to minimize Equation (1) by a generalized linear model [18]. Kernel methods replace f(x) by $w^{T}\phi (x)$, where ϕ(x) is a nonlinear mapping to a high-dimensional feature in Hilbert space ${ℋ}_{K}$. For kernel minimum mean squared error estimation problems, the optimization problem involves solving for w as in Equation (2):

(2) $$w^{*}=\arg\underset{w}{\min}\frac{1}{m}\sum_{i=1}^{m}{\left(y_{i}-w^{T}\phi (x_{i})\right)}^{2}+\lambda \left\|w\right\|$$

where a regularized term is added that penalizes large values of w and prevents the solution from overfitting the training examples. The value of λ makes a tradeoff between the minimization of the empirical risk and the smoothness of the obtained solution. Because the loss function in Equation (2) is convex, the Representer theorem [18] states that the optimal solution can be expressed as a linear combination of the training examples $w^{*}=\sum_{i=1}^{m}\alpha_{i}\phi (x_{i})$, and it can also be written as $f^{*}(x)=\sum_{i=1}^{m}\alpha_{i}\phi^{T}(x_{i})\cdot \phi (x)$. When $\phi^{T}(x_{i})\cdot \phi (x)$ is replaced by kernel $k(x_{i},x)$, the reformulation of the optimal solution that is the most common form is shown in Equation (3):

(3) $$f^{*}(x)=\sum_{i=1}^{m}\alpha_{i}k(x_{i},x)$$

where k(⋅,⋅) is a kernel function selected as a similarity measure parameter that greatly simplifies the calculation of obtaining a nonlinear model with no need for the explicitly nonlinear mapping. Here, $k(x_{i},x)$ denotes the similarity measure between training example $x_{i}$ and the new example x, and $\alpha_{i}\in ℜ,\;\forall i\in \{1,...,m\}$ is the coefficient of $k(x_{i},x)$. In Equation (3), the prediction of the new example x depends on all the training examples. The regularization term of Equation (2) can be written as a function of the coefficients $\alpha_{i}$ in Equation (3). A popular choice of regularization term is ridge regression, i.e., the $\ell_{2}$ norm that is widely used in many optimization problems; however, it cannot obtain a sparse solution. Another choice is the $\ell_{1}$ norm that has been widely studied and applied owing to its sparse characteristics.

### 2.2. Alternating Direction Method of Multipliers

Consider the separable problem in Equation (4) [29,30]:

(4) $$\begin{array}{l} \min\;\sum_{i=1}^{N}F_{i}(x_{i})\\ s.t.\;\;\;\;\;x_{i}=z,\;\;\;\;\;\;i=1,...,N\\ \;\;\;\;\;\;\;x_{i}\in P_{i},\;\;\;\;i=1,...,N \end{array}$$

where $F_{i}:{ℜ}^{m}\mapsto ℜ,\;i=1,...,N$ is a convex function, $P_{i},\;i=1,...,N$ represents the bounded polyhedral subsets of ${ℜ}^{m}$, $x_{i}\in {ℜ}^{m},\;i=1,...,N$ is a local variable, and $z\in {ℜ}^{m}$ is a global variable. Because the constraint $x_{i}=z,\;\;\;\;\;\;i=1,...,N$ is that all the local variables should be consistent or equal, this problem is called the global consensus problem. The alternating direction method of multipliers (ADMM) for Problem (4) can be derived from the augmented Lagrange method shown as Equation (5):

(5) $$L_{\rho }(x_{1},...,x_{N},z,y)=\sum_{i=1}^{N}\left(F_{i}(x_{i})+y_{i}^{T}(x_{i}-z)+(\rho /2){\left\|x_{i}-z\right\|}_{2}^{2}\right)$$

where y is the vector of dual variable or Lagrange multiplier, and $y_{i}$ is an element of y that is the dual variable on the constraint $x_{i}=z$. The ρ>0 is called the *penalty parameter*, and the quadratic term is included to overcome the lack of strict convexity of the primal function in (4). The resulting ADMM algorithm is as follows:

(6) $$x_{i}^{k+1}=\underset{\;\;\;\;\;\;\;\;\;\;\;\;\;\;x_{i}}{\arg\min}(F_{i}(x_{i})+y_{i}^{kT}(x_{i}-z)+(\rho /2){\left\|x_{i}-z^{k}\right\|}_{2}^{2})$$

(7) $$z^{k+1}=\frac{1}{N}\sum_{i=1}^{N}\left(x_{i}^{k+1}+(1/\rho )y_{i}^{k}\right)$$

(8) $$y_{i}^{k+1}=y_{i}^{k}+\rho (x_{i}^{k+1}-z^{k+1})$$

where Equations (6) and (8) are carried out independently for each i=1,...,N, and the update of global variable z in Equation (7) is handled at the central fusion center. Therefore, the ADMM algorithm operates in cycles and is considered a highly parallelizable method which applies to convex separable problems that are not necessarily strictly convex. 

### 2.3. Problem Statement

Consider a WSN with J sensor nodes. Assume that the WSN is connected and that any node j∈J only communicates with its one-hop neighboring nodes. The set of all the one-hop neighboring nodes of node j (and j itself) is denoted as $B_{j}\subseteq J$. The training example set on node j∈J is a subset of the entire training example set, which is denoted by $S_{j}:=\{(x_{jn},y_{jn}),\;\;\;\;\;n=1,2,\cdot \cdot \cdot ,N_{j}\}$, where $N_{j}$ is the number of training examples of $S_{j}$. We assume that no training examples have to be shared between neighboring nodes. To decrease the communication cost of the centralized learning method for kernel machines in WSNs, this paper studies the distributed learning method for the KMSE machine. Our research idea is to obtain a sparse model on each node by involving the $\ell_{1}$ norm regularization to reduce the amount of data transmitted between neighboring nodes. Moreover, each node communicates only with its one-hop neighboring nodes to save energy; thus, we only consider the in-network processing and the collaboration between one-hop neighboring nodes.

For ease of description, two definitions are provided.

**Definition 1.**  *(Key Samples): The training examples that correspond to the nonzero components of the coefficient vector in the optimal model are called key examples*.

**Definition 2.**  *(Sparse Rate of Model): The rate of the number of key examples to that of all the training examples used for model training is defined as the sparse rate of model*.

## 3. Distributed $\ell_{1}$-Regularized KMSE Machine

In this section, we first detail the derivation and solution of the optimization problem for the $\ell_{1}$-regularized KMSE machine. Then, we introduce the collaboration method between neighboring nodes, and, finally, propose and describe the distributed learning algorithm for the $\ell_{1}$-regularized KMSE machine.

### 3.1. Derivation and Solution of the $\ell_{1}$-Regularized KMSE Machine

The KMSE estimation problem involving the $\ell_{1}$-regularized term can be written as shown in (9):

(9) $$\underset{f\in {ℋ}_{K}}{\min}\;\;\;\;\;\;\;\;\frac{1}{m}\sum_{i=1}^{m}{\left(y_{i}-f(x_{i})\right)}^{2}\;+\;\;\;\lambda {\left\|f\right\|}_{1}$$

where $\lambda {\left\|f\right\|}_{1}$ is the $\ell_{1}$-regularized term, and λ>0 is a scalar regularized parameter usually chosen through cross-validation. If ${\left\{S_{j}\right\}}_{j=1}^{J}$ is centrally available at the fusion center of the WSN, the global optimal model $f^{*}(x)$ can be obtained by solving the optimization problem (10):

(10) $$\underset{f\in {ℋ}_{K}}{\min}\;\;\;\;\;\;\;\;\frac{1}{\sum_{j=1}^{J}N_{j}}\sum_{j=1}^{J}\sum_{n=1}^{N_{j}}\left(y_{jn}-f(x_{jn})\right){\;}^{2}\;+\;\;\;\;\;\lambda {\left\|f\right\|}_{1}\;\;,\;\;\;\;\;\;\;\;\forall j\in J,\;\;\;n=1,...,N_{j}$$

where the optimization problem (10) is the equivalent reformulation of (9); therefore, the optimization problem in (9) and (10) can obtain the same global optimal model on the same training set. Based on the form of (10), the equivalent reformulation of (10), which is easy to decompose, is constructed and written as (11):

(11) $$\underset{f\in {ℋ}_{K}}{\min}\;\;\;\;\;\;\;\;\sum_{j=1}^{J}(J/\sum_{j=1}^{J}N_{j}\sum_{n=1}^{N_{j}}\left(y_{jn}-f(x_{jn})\right){\;}^{2}\;+\;\;\;\lambda {\left\|f\right\|}_{1})\;\;,\;\;\;\;\;\;\;\;\forall j\in J,\;\;\;n=1,...,N_{j}\;$$

where $J/\sum_{j=1}^{J}N_{j}$ is a constant and can be simplified as $1/N_{j}$ when each node has the same number of training examples. The number of training examples, unless otherwise noted, is the same for each node. In (11), $\frac{1}{N_{j}}\sum_{n=1}^{N_{j}}{\left(y_{jn}-f(x_{jn})\right)}^{2}+\lambda {\left\|f\right\|}_{1}$ is referred to as the j-th term of the objective that relies only on the local training examples of node j; thus, it can be split into J subproblems that are constructed on each node with its local training examples. Our goal is to solve the problem (11) in such a way that each term can be handled by its own processor on each node. To this end, the optimization problem (11) can be rewritten with the local model $f_{j}(x)$ and a common global model f(x), shown as (12):

(12) $$\begin{array}{l} \underset{f_{j}\in {ℋ}_{K}}{\min}\;\;\;\;\;\;\sum_{j=1}^{J}\{\frac{1}{N_{j}}\sum_{n=1}^{N_{j}}\left(y_{jn}-f_{j}(x_{jn})\right){\;}^{2}\;+\;\;\;\;\;\lambda {\left\|f_{j}\right\|}_{1}\;\;\}\\ \;\;\;\;\;s.t.\;\;\;\;\;\;\;\;f_{j}\;(x_{jn})\;=f(x_{jn})\;\;,\;\;\;\;\;\;\;\;\;\forall j\in J,\;\;\;n=1,...,N_{j}\; \end{array}$$

where the equality constraint indicates that all the local models should result in the same prediction on the same training example. This approach ensures the consistency of the local model $f_{j}(x)$ and the global model f(x) on the same training set. However, $\lambda {\left\|f_{j}\right\|}_{1}$ in (12) and $\lambda {\left\|f\right\|}_{1}$ in (11) may not have the same value because different training examples are used. Therefore, the optimization problem in (12) is an approximation of the optimization problem in (11).

The equality constrained convex optimization problem (12) is typically tackled by solving its dual variable. To solve this optimization problem, the ADMM algorithm is used. The augmented Lagrangian function for problem (12) is given by Equation (13) [30]:

(13) $$\begin{array}{l} L(f_{j},f,p_{j})=\sum_{j=1}^{J}(\frac{1}{N_{j}}\sum_{n=1}^{N_{j}}{\left(y_{jn}-f_{j}(x_{jn})\right)}^{2}+\lambda {\left\|f_{j}\right\|}_{1}+p_{j}^{T}(f_{j}(x_{jn})\;-f(x_{jn}))+\frac{c}{2}{\left\|f_{j}(x_{jn})\;-f(x_{jn})\right\|}_{2}^{2})\\ \;\;\;\;\;\;\;\;\;\;\;\;\;\;\;\;\;\;\;\;\;\;\;\;\;\;\;\;\;\;\;\;\;\;\;\;\;\;\;\;\;\;\;\forall j\in J,n=1,...,N_{j} \end{array}$$

where $p_{j}$ is the dual variable or Lagrange multiplier corresponding to the equality constraint $f_{j}\;(x_{jn})\;=f(x_{jn})\;\;,\;\;\;\;\;\;\;\;\;\forall j\in J,\;\;\;n=1,...,N_{j}$, and c>0 is called the penalty parameter. To obtain the minimum value of $L(f_{j},f,p_{j})$, we take the partial derivatives of $L(f_{j},f,p_{j})$ with respect to $f_{j}$, f, and $p_{j}$, respectively, and set them to zero. This resulting ADMM algorithm is as follows Equations (14)–(16):

(14) $$f_{j}^{k+1}(x_{jn}):=\arg\underset{f_{j}}{\min}(\frac{1}{N_{j}}\sum_{n=1}^{N_{j}}{\left(y_{jn}-f_{j}(x_{jn})\right)}^{2}+\lambda {\left\|f_{j}\right\|}_{1}+p_{j}^{kT}(f_{j}(x_{jn})-f^{k}(x_{jn}))+\frac{c}{2}{\left\|f_{j}(x_{jn})-f^{k}(x_{jn})\right\|}_{2}^{2})$$

(15) $$f^{k+1}(x_{jn}):=\frac{1}{J}\sum_{j=1}^{J}\left(f_{j}^{k+1}(x_{jn}\right)+(1/c)\;p_{j}^{k})$$

(16) $$p_{j}^{k+1}=p_{j}^{k}+c(f_{j}^{k+1}(x_{jn})-f^{k+1}(x_{jn}))$$

where Equations (14) and (16) are carried out independently on each node j∈1,...,J, and the global optimal model f(x) is usually handled in the central fusion center. This algorithm can be simplified further. With the average (j∈1,...,J) of a prediction denoted with an overline, the update of $f^{k+1}(x_{jn})$ can be written as Equation (17). Similarly, averaging the update of $p_{j}^{k}$ yields Equation (18):

(17) $$f^{k+1}(x_{jn})={\bar{f}}^{k+1}(x_{jn})+(1/c){\bar{p}}^{k}$$

(18) $${\bar{p}}^{k+1}={\bar{p}}^{k}+c({\bar{f}}^{k+1}(x_{jn})-f^{k+1}(x_{jn}))$$

Substituting Equation (17) into Equation (18) shows that ${\bar{p}}^{k+1}=0$, i.e., the dual variables have an average value of zero after the first iteration. Using $f^{k+1}(x_{jn})={\bar{f}}^{k+1}(x_{jn})$, the ADMM algorithm can be written as Equations (19)–(21):

(19) $$f_{j}^{k+1}(x_{jn}):=\arg\underset{f_{j}}{\min}(\frac{1}{N_{j}}\sum_{n=1}^{N_{j}}{\left(y_{jn}-f_{j}(x_{jn})\right)}^{2}+\lambda {\left\|f_{j}\right\|}_{1}+p_{j}^{kT}(f_{j}(x_{jn})-{\bar{f}}^{k}(x_{jn}))+\frac{c}{2}{\left\|f_{j}(x_{jn})-{\bar{f}}^{k}(x_{jn})\right\|}_{2}^{2})$$

(20) $${\bar{f}}^{k+1}(x_{jn})=\frac{1}{J}\sum_{j=1}^{J}f_{j}^{k+1}(x_{jn})$$

(21) $$p_{j}^{k+1}=p_{j}^{k}+c(f_{j}^{k+1}(x_{jn})-{\bar{f}}^{k+1}(x_{jn}))$$

Note that the average predictions ${\bar{f}}^{k+1}(x_{j})\;\;$ of the local training examples on node j are obtained by all models on each node within the WSN; consequently, all models are required to be on each node. However, this will significantly increase the communication cost and energy consumption of each node without relying on the fusion center or on special WSN nodes. To reduce the communication overhead, the local average predictions ${\bar{f_{B_{j}}}}^{k+1}(x_{j})$ obtained by models on single-hop neighboring nodes are used as the approximation to the global average predictions ${\bar{f}}^{k+1}(x_{j})\;\;$ that would be obtained by all models on each node. As a consequence, the iterative Formulas (19)–(21) can be rewritten as Equations (22)–(24):

(22) $$\begin{array}{l} f_{j}^{k+1}(x_{jn})=\arg\underset{f_{j}}{\min}\{\frac{1}{N_{j}}\sum_{n=1}^{N_{j}}{\left(y_{jn}-f_{j}(x_{jn})\right)}^{2}+\lambda {\left\|f_{j}\right\|}_{1}\\ \;\;\;\;\;\;\;\;\;\;\;\;\;\;\;\;\;\;\;\;\;\;\;\;\;\;\;\;\;\;\;\;\;\;\;\;\;\;\;\;\;\;\;\;\;\;\;+p_{j}^{kT}(f_{j}(x_{jn})-{\bar{f_{B_{j}}}}^{k}(x_{jn}))+\frac{c}{2}{\left\|f_{j}(x_{jn})-{\bar{f_{B_{j}}}}^{k}(x_{jn})\right\|}_{2}^{2}\} \end{array}$$

(23) $${\bar{f_{B_{j}}}}^{k+1}(x_{jn})=\frac{\sum_{j=1}^{B_{j}}f_{j}^{k+1}(x_{jn})}{\left|B_{j}\right|}$$

(24) $$p_{j}^{k+1}=p_{j}^{k}+c(f_{j}^{k+1}(x_{jn})-{\bar{f_{B_{j}}}}^{k+1}(x_{jn}))$$

where $B_{j}\subseteq J$ is the set of neighboring nodes of node j∈J, including itself; $\left|B_{j}\right|$ denotes the number of nodes in $B_{j}$; and ${\bar{f_{B_{j}}}}^{k}(x_{j})$ denotes the average predictions of local training examples on node j, which are obtained by models on nodes in $B_{j}$ at the k-th iteration. In this paper, the distributed learning algorithm for the $\ell_{1}$-regularized KMSE machine is executed through iterations over Equations (22)–(24).

The optimization problem (22) is still a non-constrained convex optimization problem that only relies on its local training examples on each node. To improve the convenience of solving this optimization problem, it is rewritten in matrix form in (25):

(25) $$\min\;\frac{1}{N_{j}}{\left(Y_{j}-K_{j}\alpha_{j}\right)}^{T}(Y_{j}-K_{j}\alpha_{j})+p_{j}^{T}(K_{j}\alpha_{j}-\bar{f_{B_{j}}}(x_{jn}))+\frac{c}{2}{\left\|K_{j}\alpha_{j}\;-\bar{f_{B_{j}}}(x_{jn})\right\|}_{2}^{2}+\lambda {\left\|\alpha_{j}\right\|}_{1}$$

where $Y_{j}\in R^{N_{j}}$ is the output vector of training examples on node j∈J, $K_{j}\in R^{N_{j}\times (N_{j}+1)}$ is the augmented matrix obtained by $k(x_{1}\cdot x_{2}),\;\;\;\forall \;x_{1},x_{2}\in S_{j}$, and $\alpha_{j}\in R^{N_{j}+1}$ is the coefficient vector. To solve the non-constrained optimization problem in (25), an equivalent reformulation of (25) with an equality constraint is constructed as shown in (26). Then, the ADMM is used to solve the optimization problem in (26), and the resulting iterations are shown in Equations (27)–(29):

(26) $$\begin{array}{l} \min\;\frac{1}{N_{j}}{\left(Y_{j}-K_{j}\alpha_{j}\right)}^{T}(Y_{j}-K_{j}\alpha_{j})+p_{j}^{T}(K_{j}\alpha_{j}-\bar{f_{B_{j}}}(x_{jn}))+\frac{c}{2}{\left\|K_{j}\alpha_{j}-\bar{f_{B_{j}}}(x_{jn})\right\|}_{2}^{2}+\lambda {\left\|z_{j}\right\|}_{1}\\ \;\;\;\;\;\;\;\;\;\;\;\;\;\;\;\;\;s.t.\;\;\;\;\;\;\;\;\;\;\;\;\;\;\;\alpha_{j}-z_{j}=0\; \end{array}$$

(27) $$\alpha_{j}^{k+1}:={\left[(\frac{2}{N_{j}}+c)K_{j}^{T}K_{j}+\rho I\right]}^{-1}\;\;[K_{j}^{T}(\frac{2}{N_{j}}Y_{j}+c{\bar{f_{B_{j}}}}^{k}(x_{j})-p_{j}^{k})+\rho (z_{j}^{k}+u_{j}^{k})]$$

(28) $$z_{j}^{k+1}:=S_{\lambda /\rho }(\alpha_{j}^{k+1}+u_{j}^{k})$$

(29) $$u_{j}^{k+1}:=u_{j}^{k}+\alpha_{j}^{k+1}-{z_{j}}^{k+1}$$

In Equation (27), I is the identity matrix, and $(\frac{2}{N_{j}}+c)K_{j}^{T}K_{j}+\rho I$ is always invertible because ρ>0. In Equation (28), S is the soft thresholding operator, which is defined as shown in Equation (30):

(30) $$S_{k}(a)=\{\begin{array}{cc} a-k & a>k\\ 0 & \left|a\right|\leq k\\ a+k & a<-k \end{array}$$

Now, each node j∈J can obtain the sparse coefficient vector $a_{j}$ through the iterations from Equations (27) to (29); that is, there are relatively few key examples in the model. The model obtained for each node can be expressed as in Equation (31):

(31) $${f_{j}}^{k+1}(x)=\sum_{i=1}^{l}{\alpha_{ji}}^{k+1}\;k(x_{ji}\cdot x)\;\;,\;\;\;\forall j\in J,\;\;\;n=1,...,N_{j}$$

where the form of Equation (31) is the same as that of Equation (3), l is the number of key examples, and $a_{ji}$ is a nonzero value.

Consequently, the distributed learning algorithm for the $\ell_{1}$-regularized KMSE machine can be executed in the sequence Equations (27)–(29), (23), (24) for each iteration until each node obtains a stable model.

### 3.2. Collaboration Method between Neighboring Nodes

In Equation (23), the average predictions ${\bar{f_{B_{j}}}}^{k+1}(x_{j})$ of the training examples on node j∈J are the average of the predictions of the local training examples predicted by models on its single-hop neighboring nodes in $B_{j}$. To obtain the average predictions ${\bar{f_{B_{j}}}}^{k+1}(x_{j})$, each node requires all the models on its single-hop neighboring nodes. Therefore, transferring the sparse models between single-hop neighboring nodes is required. After each node receives all the models from its neighboring nodes, all the key examples in these models are added to the local training example set, and, then, all the received models are used to predict the average predictions of all the local training examples. In short, the distributed learning method presented here depends on in-network processing, with sparse models transferred only between single-hop neighboring nodes.

### 3.3. Distributed Learning Algorithm for the $\ell_{1}$-Regularized KMSE Machine

Based on the derivation and solution of the $\ell_{1}$-regularized KMSE estimation problem and the collaboration between neighboring nodes, we propose a distributed learning algorithm for the $\ell_{1}$-regularized KMSE machine (L1-DKMSE). The detailed steps of L1-DKMSE are illustrated as follows: **Algorithm 1:** L1-DKMSE  
**Input**: Initialize the training set $S_{j}:=(x_{j},y_{j})$ for node j∈J, iterations k=0, ${\bar{f_{B_{j}}}}^{k}(x_{j})=y_{j}$, and $p_{j}^{k}(x_{j})=0$, choose the Radial Basis Function (RBF) as the kernel function, and initialize the kernel parameter σ and the regularization parameter λ.  
**Output**: the sparse model $f_{j}^{*}(\cdot ),\forall j\in J$.  
**Repeat:**  
**Step 1:** each node j∈J obtains its sparse model $f_{j}^{k}(\cdot )$ by iterations of Equations (27)–(29) using its training examples. Then, it broadcasts its sparse model to its one-hop neighboring nodes in $B_{j}$.  
**Step 2:** each node j∈J receives $f_{i}^{k}(\cdot ),i\in B_{j}$ and adds the key examples in $f_{i}^{k}(\cdot ),i\in B_{j}$ to its local training set.  
**Step 3:** each node j∈J predicts its local training examples by using $f_{i}^{k}(\cdot ),\;i\in B_{j}$ and then computes ${\bar{f_{B_{j}}}}^{k}(x_{j})$ and $p_{j}^{k}(x_{j})$ using Equations (23) and (24), respectively.  
**Step 4:** If the models $f_{j}^{k}(\cdot )$ on each node are all stable, stop; otherwise, increment k (k=k+1) and return to **Step 1**.

## 4. Numerical Simulations

To analyze the performance of Algorithm 1, extensive simulation experiments have been conducted using a synthetic dataset and UCI datasets. Consider a randomly generated network with J=30 nodes connected with a minimum degree per node of two and a maximum degree per node of five. The Centralized L1-regularized KMSE learning algorithm (L1-CKMSE), the Centralized SVM learning algorithm (CSVM), the Distributed Kernel Least Squares learning algorithm (DKLS) in [10], the Distributed Parallel SVM learning algorithm (DPSVM) in [17], and the Distributed SVM learning algorithm based on a nonlinear kernel (NDSVM) in [14] are compared with Algorithm 1 with regard to prediction accuracy, sparse rate of model, communication cost, and iterations.

### 4.1. Synthetic Dataset

**Figure 1:**
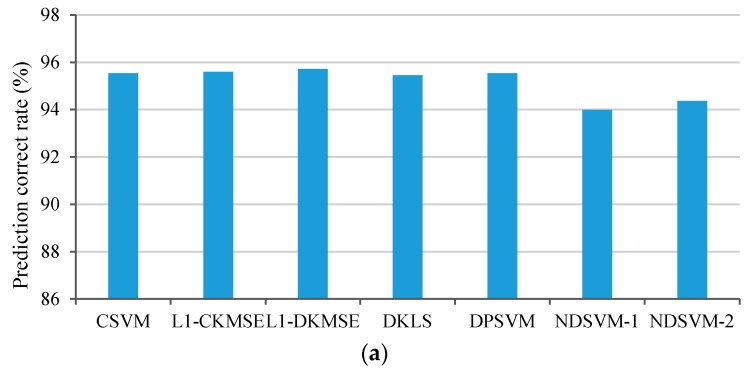

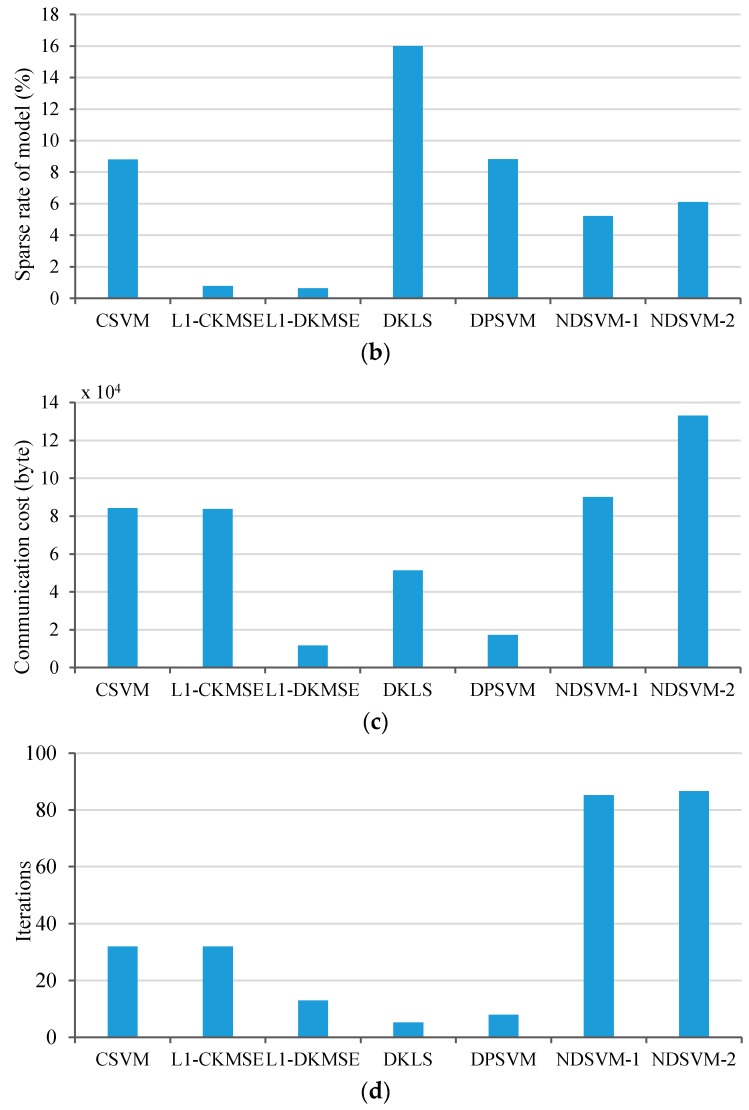
Performance comparisons of CSVM, L1-CKMSE, L1-DKMSE, DKLS, DPSVM and NDSVM on the synthetic dataset: (**a**) prediction accuracy; (**b**) sparse rate of model; (**c**) communication cost; and (**d**) iterations.

The synthetic dataset is composed of the labeled training examples from two different equiprobable, nonlinear, separable classes $C_{1}$ and $C_{2}$. Class $C_{1}$ contains examples from a two-dimensional Gaussian distribution with a covariance matrix of Σ=[0.6,0;0,0.4] and a mean vector $mu_{1}={\left[0,0\right]}^{T}$. Class $C_{2}$ is a mixture of Gaussian distributions with the mixing parameters $\pi_{21}=0.3$ and $\pi_{22}=0.7$, the mean vectors $mu_{2}={\left[-2,-2\right]}^{T}$ and $mu_{3}={\left[2,2\right]}^{T}$, and the equal covariance matrix Σ. Each local training set $S_{j}$ consists of $N_{j}=N=60$ labeled examples that are randomly generated from the distributions described above; the number of examples per class is equivalent. Thus, the number of all training examples is JN=1800. Similarly, 500 examples per class are randomly generated as test examples. The Gaussian kernel function was used to construct the global nonlinear classifier in this simulation. Because the concept underlying each algorithm is specific to that algorithm, the optimal value of the parameters in each algorithm is different from that in the other algorithms. The optimal values of σ and C in CSVM, DPSVM and NDSVM are chosen through cross-validations; thus, as a result, σ=0.25,C=2.0 are used in these three algorithms. The optimal values of σ and λ in L1-CKMSE, L1-DKMSE and DKLS are selected by using the grid search method, and the result is σ=0.2,λ=1.2. The NDSVM algorithm requires preselected examples common to all nodes. To demonstrate the impact of the number of shared examples on the performance of the algorithm, 30 and 50 shared examples were used, and the corresponding experimental results were labeled NDSVM-1 and NDSVM-2, respectively. Based on the experimental setup described above, 30 independent runs were performed. The simulation results of the performance comparison in terms of the prediction accuracy, sparse rate of model, communication cost, and iterations are shown in Figure 1.

In general, the prediction accuracy obtained by the centralized learning algorithm is used as the benchmark. Thus, the prediction accuracy of the L1-CKMSE algorithm is used as the benchmark. As shown in Figure 1a, the prediction accuracy of CSVM, L1-CKMSE, DKLS, DPSVM, and L1-DKMSE algorithms on the synthetic dataset is nearly equivalent; however, NDSVM-1 and NDSVM-2 obtain a relatively low prediction accuracy. Therefore, the prediction accuracy of L1-DKMSE algorithm is nearly equivalent to the benchmark on the synthetic dataset and is much better than that of NDSVM.

The sparse rate of model is the ratio of the number of key examples to that of all training examples (see Definitions 1 and 2); thus, a lower sparse rate of model or fewer key examples is better. As shown in Figure 1b, the sparse rate of model obtained by CSVM, DKLS, DPSVM and NDSVM on the synthetic dataset is significantly higher than that obtained by L1-CKMSE and L1-DKMSE, whereas the sparse rate of model obtained by L1-CKMSE is slightly higher than that obtained by L1-DKMSE. Specifically, the sparse rate of model obtained by L1-CKMSE is 19.75% higher than that obtained by L1-DKMSE on the synthetic dataset. A comparison of the sparse rate of model obtained by all the compared algorithms shows that our proposed L1-DKMSE algorithm significantly outperforms the compared algorithms in this respect, indicating that L1-DKMSE can obtain the sparsest model among all the compared algorithms. Therefore, our algorithm can significantly reduce the computing costs of performing predictions.

The communication costs are measured in terms of the number of scalars transmitted on all nodes. As shown in Figure 1c, the communication cost for L1-DKMSE is significantly less than that for CSVM, L1-CKMSE, DKLS, and NDSVM, and it is close to that for DPSVM on the synthetic dataset. Specifically, the communication costs for L1-DKMSE are 85.53%, 85.46%, 77.56%, 87.06%, and 91.24% less than for CSVM, L1-CKMSE, DKLS, NDSVM-1 and NDSVM-2, respectively, and 27.85% less than for DPSVM. Consequently, L1-DKMSE has been shown to significantly outperform CSVM, L1-CKMSE, DKLS, DPSVM and NDSVM in terms of communication cost on the synthetic dataset.

As shown in Figure 1d, the iterations required by L1-DKMSE are slightly higher than those required by DKLS and DPSVM but significantly less than those required by CSVM, L1-CKMSE, NDSVM-1 and NDSVM-2. These results show that our proposed L1-DKMSE requires relatively few iterations on the synthetic dataset.

### 4.2. UCI Datasets

To further verify the applicability and effectiveness of the L1-DKMSE algorithm, three datasets from the UCI repository are used to conduct experiments. Brief descriptions of these three datasets are listed in Table 1. From each dataset, 900 examples per class are randomly chosen as training examples, and 500 examples per class are chosen as test examples. For simulation purposes, all the training examples are randomly assigned to each node of the given network, and each node has exactly the same number of examples from each class.

Table 2 shows the optimal values of the parameters used in the different algorithms for each dataset. The optimal values of the parameters used in CSVM, DPSVM and NDSVM are chosen by cross-validations, and those in L1-CKMSE, L1-DKMSE and DKLS are selected by the grid search method. Moreover, the number of shared examples in the NDSVM algorithm is represented as *L*.

**Figure 2:**
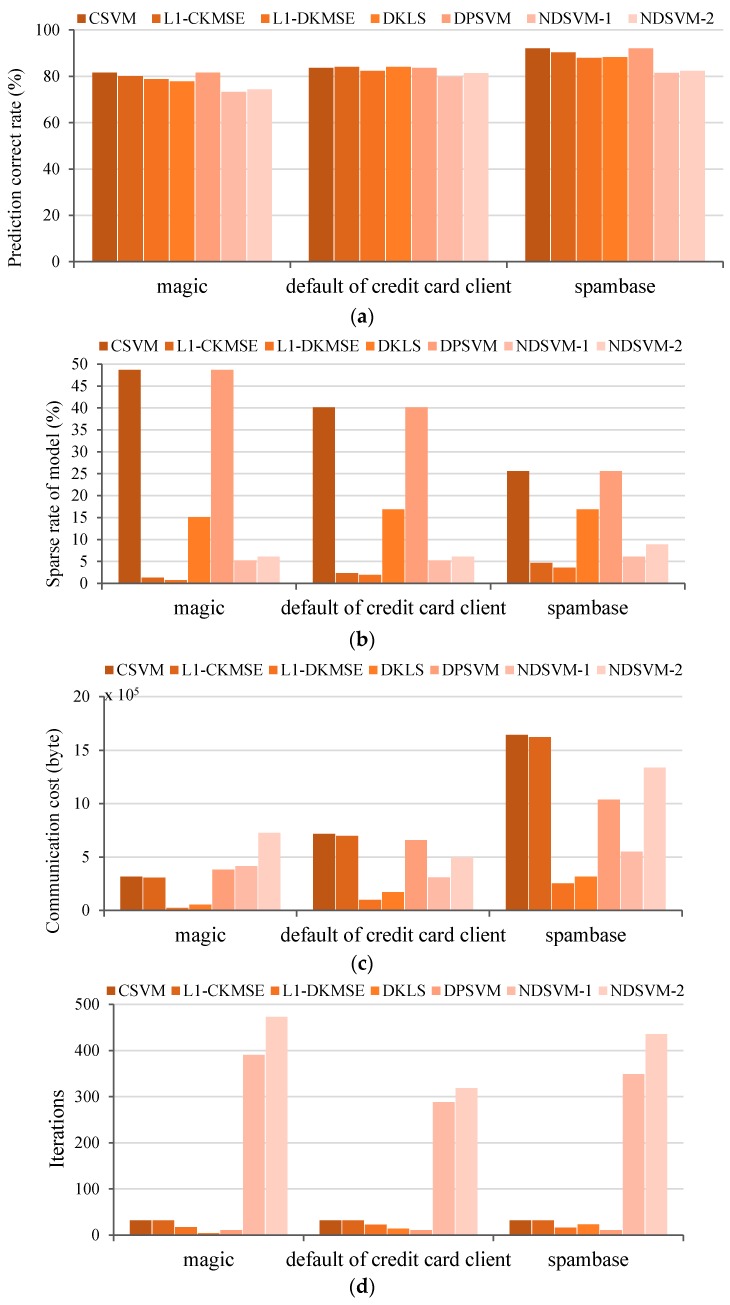
Performance comparisons of CSVM, L1-CKMSE, L1-DKMSE, DKLS, DPSVM and NDSVM on the UCI datasets: (**a**) prediction accuracy; (**b**) sparse rate of model; (**c**) communication cost; and (**d**) iterations.

Based on the experimental setup shown in Table 1 and Table 2, 30 independent runs were performed on each dataset. The simulation results are shown in Figure 2.

In Figure 2a, the prediction accuracy achieved of CSVM, L1-CKMSE, and DPSVM is almost the same for each dataset. The prediction accuracy of L1-DKMSE is slightly below that obtained by the centralized learning algorithms (L1-CKMSE and CSVM) on the same dataset, but slightly higher than that obtained by NDSVM on the same dataset. Specifically, the prediction accuracy obtained by L1-DKMSE is 1.61%, 2.12%, and 2.64% below the prediction accuracy obtained by L1-CKMSE on the magic, default of credit card client and spambase datasets, respectively. The comparison shows that no distinct differences in terms of prediction accuracy occurred between L1-DKMSE and L1-CKMSE, indicating that the prediction accuracies of L1-DKMSE and L1-CKMSE are nearly equivalent for each dataset.

As shown in Figure 2b, the sparse rate of model obtained by CSVM, DKLS, DPSVM and NDSVM on each dataset is significantly higher than that obtained by L1-CKMSE and L1-DKMSE for the same dataset, whereas the sparse rate of model obtained by L1-CKMSE is much higher than that obtained by L1-DKMSE. Specifically, the sparse rate of model results obtained by L1-DKMSE are 45.04%, 16.81%, and 22.96% lower than those obtained by L1-CKMSE on the magic, default of credit card client and spambase datasets, respectively. A comparison of the sparse rate of model obtained by these algorithms shows that the L1-DKMSE algorithm significantly outperforms the compared algorithms, indicating that L1-DKMSE can obtain the sparsest model among all the algorithms tested in this simulation and can significantly reduce the computing costs for performing predictions.

As shown in Figure 2c, the communication costs for L1-DKMSE are significantly lower than those for CSVM, L1-CKMSE, DKLS, DPSVM and NDSVM on each dataset. Among the five comparison algorithms, the communication costs for DKLS are the closest to those for the L1-DKMSE algorithm; however, the communication costs for L1-DKMSE are 57.02%, 41.69% and 19.71% less than those for DKLS on the magic, default of credit card client and spambase datasets, respectively. The comparison results show that L1-DKMSE significantly outperforms the compared algorithms with respect to communication costs on each dataset listed in Table 1.

As shown in Figure 2d, the iterations of L1-DKMSE are slightly higher than those of DKLS and DPSVM on the magic and default of credit card client datasets and slightly higher than those of DPSVM on the spambase dataset but significantly less than those of CSVM, L1-CKMSE and NDSVM. The simulation results of these three datasets of UCI show that our proposed L1-DKMSE algorithm requires relatively fewer iterations.

## 5. Experiment on Test Platform

To further compare the communication costs of the different algorithms, an experiment is conducted on a test platform developed by our team to validate the proposed distributed learning method for kernel methods. In this experiment, the experimental results for the communication costs on the synthetic dataset and only the communication costs of sending data are considered. Thus, the average amount of data at every turn on each node is easily calculated. The average amount of data sent at every turn on each node and the iterations of the different algorithms are shown in Table 3.

Two 18650-type Li-ion batteries are used to power the sensor node. The direct load method is used to calculate the battery capacity from the battery voltage. The correspondence of voltage and battery capacity is shown in Table 4.

**Figure 3:**
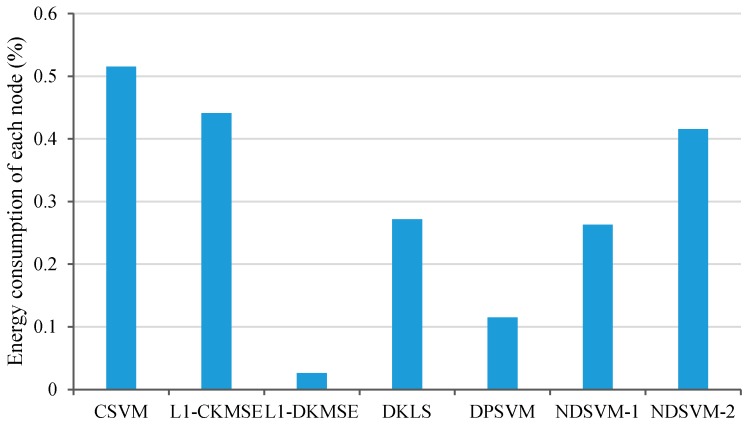
Comparison of the energy consumption on each node for the CSVM, L1-CKMSE, L1-DKMSE, DKLS, DPSVM and NDSVM algorithms on the test platform.

For each of the different algorithms, each node broadcasts a certain amount of data at every turn and repeats the iterations listed in Table 3. The energy consumption of each node for the different algorithms can be calculated by the relationship between voltage and battery capacity in Table 4. The experimental results of the energy consumption of each node for the different algorithms are illustrated in Figure 3. As Figure 3 shows, the energy consumption of each node for L1-DKMSE is significantly less than that for CSVM, L1-CKMSE, DKLS, DPSVM and NDSVM. Among the five comparison algorithms, the energy consumption of each node for the DPSVM algorithm is closest to that of our proposal; however, the energy consumption of each node for L1-DKMSE is still 77.16% less than that for DPSVM. The comparison results show that L1-DKMSE significantly outperforms the compared algorithms in terms of the energy consumption of each node.

## 6. Conclusions

In this paper, we presented a distributed learning algorithm, L1-DKMSE, for the $\ell_{1}$-regularized KMSE machine and demonstrated its effectiveness through simulation experiments as well as a test platform experiment. The experimental results indicated that L1-DKMSE can obtain almost the same prediction accuracy as that of the centralized learning method and learn a very sparse model. In particular, it can significantly decrease the communication costs during the model training process and can converge with fewer iterations. Because of its remarkable advantages in terms of the communication cost and the sparseness of the model, the L1-DKMSE algorithm is considered a feasible learning method for kernel machines in WSNs. In future work, we will explore the following topics: (1) how to transmit and share training examples between neighboring nodes under unreliable communication links; (2) how to select a neighboring node according to the residual energy of its neighbor nodes; and (3) how to apply an online learning method for a kernel machine to reduce the computational complexity and memory requirements.

## Figures and Tables

**Table 1:** Datasets from UCI repository.

Datasets	Classes	Dim. Feature	Size
magic	2	10	19,020
default of credit card client	2	24	30,000
spambase	2	57	4601

**Table 2:** Values of the parameters of the algorithms on the UCI datasets. CSVM, Centralized SVM learning algorithm; L1-CKMSE, Centralized L1-regularized KMSE learning algorithm; L1-DKMSE, proposed Algorithm 1 in this paper; DKLS, Distributed Kernel Least Squares learning algorithm in [10]; DPSVM, Distributed Parallel SVM learning algorithm in [17]; NDSVM-1, Distributed SVM learning algorithm based on a nonlinear kernel in [14] with 30 shared training examples among all nodes; NDSVM-2, Distributed SVM learning algorithm based on a nonlinear kernel in [14] with 50 shared training examples among all nodes.

Algorithms	Magic	Default of Credit Card Client	Spambase
CSVM	σ=0.7,C=16	σ=0.5,C=16	σ=1.0,C=16
L1-CKMSE	σ=1.1,λ=0.3	σ=1.0,λ=0.6	σ=1.0,λ=0.4
L1-DKMSE	σ=1.1,λ=0.3	σ=1.0,λ=0.6	σ=1.0,λ=0.4
DKLS	σ=1.7,λ=0.3	σ=2.0,λ=1.0	σ=2.0,λ=1.0
DPSVM	σ=0.7,C=16	σ=0.5,C=16	σ=1.0,C=16
NDSVM-1	σ=0.7,C=2,L=30	σ=1.4,C=2,L=30	σ=0.8,C=2,L=50
NDSVM-2	σ=0.7,C=2,L=50	σ=1.4,C=2,L=30	σ=0.8,C=2,L=100

**Table 3:** Communication costs of the algorithms for the synthetic dataset.

Algorithms	Amount of Data Sent at Every Turn of Each Node (Byte)	Iterations
CSVM	140	20
L1-CKMSE	140	20
L1-DKMSE	26	16
DKLS	312	6
DPSVM	67	9
NDSVM-1	35	85
NDSVM-2	51	87

**Table 4:** Correspondence of voltage and battery capacity.

500 mA Load Method												
Voltage (V)	8.40	7.94	7.74	7.58	7.46	7.36	7.30	7.24	7.16	7.02	6.84	6.00
Battery capacity (%)	100	90	80	70	60	50	40	30	20	10	5	0
